# Identification and analysis of differentially expressed trihelix genes in maize (*Zea mays*) under abiotic stresses

**DOI:** 10.7717/peerj.15312

**Published:** 2023-05-01

**Authors:** Dongbo Zhao, Fengju Gao, Peiyan Guan, Jiansheng Gao, Zhihui Guo, Jianjun Guo, Huini Cui, Yongjun Li, Guijun Zhang, Zhao Li, Lianghai Guo

**Affiliations:** 1 Dezhou Academy of Agricultural Science, Dezhou, Shandong, China; 2 Dezhou University, Dezhou, Shandong, China

**Keywords:** Bioinformatics analysis, Trihelix transcription factor, Expression profiles, Heat stress, Drought stress

## Abstract

**Background:**

Trihelix transcription factors play important roles in triggering plant growth and imparting tolerance against biotic and abiotic stresses. However, a systematical analysis of the trihelix transcription factor family under heat and drought stresses in maize has not been reported.

**Methods:**

PlantTFDB and TBtools were employed to identify the trihelix domain-containing genes in the maize genome. The heat-regulated transcriptome data for maize were obtained from NCBI to screen differentially expressed *ZmTHs* genes through statistical analysis. The basic protein sequences, chromosomal localization, and subcellular localization were analyzed using Maize GDB, Expasy, SOMPA, TBtools, and Plant-mPLoc. The conserved motifs, evolutionary relationships, and *cis*-elements, were analyzed by MEME, MEGA7.0 and PlantCARE software, respectively. The tissue expression patterns of *ZmTHs* and their expression profiles under heat and drought stress were detected using quantitative real-time PCR (qRT-PCR).

**Results:**

A total of 44 trihelix family members were discovered, and members were distributed over 10 chromosomes in the maize genome. A total of 11 genes were identified that were regulated by heat stress; these were unevenly distributed on chromosomes 1, 2, 4, 5, and 10. *ZmTHs* encoded a total of 16 proteins, all of which were located in the nucleus; however, ZmTH04.1 was also distributed in the chloroplast. The protein length varied from 206 to 725 amino acids; the molecular weight ranged from 22.63 to 76.40 kD; and the theoretical isoelectric point (pI) ranged from 5.24 to 11.2. The protein’s secondary structures were mainly found to be random coils and α-helices, with fewer instances of elongation chains and β-rotations. Phylogenetic relationship analysis showed that these can be divided into five sub-groups. The conserved domain of *ZmTHs* was GT1 or MyB_DNA-Bind_4. The protein and gene structure of *ZmTHs* differed greatly among the subfamilies, while the structures within the subfamilies were similar. The promoter of *ZmTHs* contained abundant tissue-specific expression *cis*-acting elements and abiotic stress response elements. qRT-PCR analysis showed that *ZmTHs* expression levels were significantly different in different tissues. Furthermore, the expression of *ZmTH08* was dramatically up-regulated by heat stress, while the expression of *ZmTH03*, *ZmTH04*, *ZmTH05*, *ZmTH06*, *ZmTH07*, *ZmTH09*, *ZmTH10*, and *ZmTH11* were down-regulated by heat stress. Upon PEG-simulated drought stress, *ZmTH06* was significantly up-regulated, while *ZmTH01* and *ZmTH07* were down-regulated.

**Conclusions:**

We performed a genome-wide, systematic identification and analysis of differentially expressed trihelix genes under heat and drought stresses in maize.

## Introduction

Plants are often subjected to stresses during their growth and development. Of these stresses, abiotic stresses, such as high temperature and drought, are the main factors causing crop yield reduction (Ashfaq et al., 2022; Zhu, 2016). Global warming causes a greater incidence of higher temperatures, especially during critical periods of maize growth and development; this adversely affects maize spikelet differentiation, flowering, pollination, and grain filling (Zenda et al., 2022). Transcription factors can improve the abiotic stress tolerance of plants by activating or repressing the expression of related genes (Oliveira et al., 2023). The trihelix transcription factors are significant for their important roles in plant growth and development, abiotic stresses, and pathogen defense (Kaplan-Levy et al., 2012; Zhang et al., 2021).

Trihelix transcription factors are a plant-specific transcription factor family, with a typical triple-helix (helix-loop-helix-loop-helix) structure. It binds GT elements in the DNA sequence and is therefore also known as a GT factor (Zhou, 1999). *GT-1* in pea (*Pisum sativum*) was the first trihelix transcription factor identified. It can bind specifically to the GT *cis*-element (Green, Kay & Chua, 1987). *GT-1* from tobacco was subsequently cloned and it was discovered that an α-helix is required for DNA binding (Lam, 1995). Meanwhile, another member of the trihelix transcription factor was described in rice with a different sequence binding preference than GT-1; this was named GT-2 (Dehesh, Bruce & Quail, 1990). Based on the structural domain characteristics, the trihelix family genes were divided into five subfamilies: GT-1, GT-2, GTγ, SH4, and SIP1 (Kaplan-Levy et al., 2012). Each subfamily contains an N-terminal trihelix conserved motif, but the C-terminal varies. Research on the trihelix transcription factor initially focused on the regulation of light-dependent target genes (Zhou, 1999). Subsequently, a number of trihelix factors were identified and cloned from Arabidopsis, rice, and other plant species. Trihelix genes have been since been shown to regulate growth and development and participate in stress responses.

*EMB2746*, *EAD31*, *SH4*, *ASIL1*, *ASIL2* and *ZmThx20* are reportedly related to embryo formation and seed development (Kaplan-Levy et al., 2012; Li et al., 2021b). *AtGT4*, *GhGT26*, *OsGTγ-1*, and *OsGTγ-2* enhance plants’ salt stress tolerance (Fang et al., 2010; Li et al., 2022; Liu et al., 2020b; Wang et al., 2014). *AtGTL1* negatively regulates water use efficiency and drought tolerance in Arabidopsis by modulating stomatal density (Yoo et al., 2010). The overexpression of *GmGT-2A*, *GmGT-2B*, *Sb06g023980*, and *Sb06g024110* can improve the tolerance of Arabidopsis to salt, low temperature, and drought stress (Qin et al., 2014; Xie et al., 2009). However, the high expression of *TaGT2L1D* in wheat can significantly increase the number of stomata and reduce the drought tolerance of plants (Zheng et al., 2016). *ShCIGT* responds to a variety of abiotic stresses and its overexpression in tomato significantly increases the plant’s tolerance to low temperature and drought stresses (Yu et al., 2018). Most trihelix genes in *Brassica napus* were significantly regulated under heat treatment (Zhang et al., 2022). Additionally, 12 *SbTHs* were significant upregulated by high temperature stress in sorghum (Li et al., 2021a). *GT-1* is required for thermotolerance and acts as a mediator, linking the signal perception and activation of the cellular thermal response by activating the expression of *HsfA2* (He et al., 2022). *ZmGT-3b* knockdown seedlings were found to improve drought tolerance (Zhang et al., 2021). These results indicate that trihelix family members play important roles in plant development and abiotic stress responses.

Maize is an important food crop worldwide, and often subjected to various stresses during growth and development, especially heat and drought stresses, which seriously affect yield and quality. Identification of functional genes related to abiotic stress in maize is important for improving resistance. Bioinformatics has been used to identify members of the maize trihelix family (Du, Huang & Liu, 2015; Jiang et al., 2020; Qin et al., 2014); however, the systematic understanding of trihelix genes in maize is still limited and its response to abiotic stress is unclear. In this study, bioinformatics was used to identify the members, classification, gene structure, chromosome location, and evolutionary relationship of the trihelix family. qRT-PCR was used to detect changes in the transcript levels of *ZmTHs* genes under heat and drought stress. This research will lay a foundation for the in-depth analysis of the biological functions of *ZmTHs* in response to abiotic stresses such as heat and drought, and provided potential genetic resources for genetic improvement of stress-tolerant maize strains.

## Materials and Methods

### Plant materials, growth conditions and stress treatments

The experiments were conducted in the Science Park of the Dezhou Academy of Agricultural Science (116.3467E, 37.3600N) and Dezhou University. Maize seeds were preserved at Dezhou Academy of Agricultural Science and Mo17 was more heat tolerant than B73 (Bheemanahalli et al., 2022; Jorgensen et al., 1992). Inbred lines of maize (*Zea mays* L.) were grown in a greenhouse at 28 °C with a photoperiod of 16 h light and 8 h dark. Seedlings of uniform growth were selected during the flowering stage for tissue expression analysis. We selected the mature root, root tip, spike internode, internode under spike, mature leaf, silks, and female spikelet. For the heat stress treatment, maize seedlings in the three-leaf stage were placed in a light incubator for 4 h at 42 °C, while the control was grown under normal conditions. The above-ground parts were collected immediately. For drought stress treatments, three-leaf-old seedlings of the inbred line B73 were immersed in 20% (w/v) polyethylene glycol 6000 (PEG-6000) solution, and were treated for 4 and 24 h, respectively. The roots of the seedlings were collected for analysis (Jiao et al., 2022; Zeng et al., 2019). Six seedlings were treated per sample, and three biological replicates were conducted for each sample. Figures S1 and S2 show the maize seedlings before and after heat and drought treatment, respectively. All samples were snap-frozen in liquid nitrogen and stored at −80 °C.

### Identification and phylogenetic analysis of trihelix family members

The trihelix protein sequences for maize were retrieved from PlantTFDB (http://planttfdb.gao-lab.org/). The trihelix family members were verified using the Pfam database (http://pfam.xfam.org/) and the CDD online tool from the NCBI (https://www.ncbi.nlm.nih.gov/cdd/). A phylogenetic tree was constructed using the neighbor-joining method (NJ) on MEGA7.0 software (Kumar et al., 2018) with bootstrap values from 1,000 replicates.

### Analysis expression profiles of trihelix family under heat stress

Genevestigator software (https://genevestigator.com/) (Hruz et al., 2008) was used to compare the maize trihelix genes against the maize GeneChip platform to analyze the transcriptional profile under heat stress. Then, maize seedling expression profiles were downloaded from the NCBI under accession number SRP106663 (Waters et al., 2017). The heat treatment process was as follows: maize seedlings (inbred lines B73, Oh43, and Oh43 × B73) were grown for 14 days under long daylight conditions in a 16/8 h light incubator, then seedlings were placed in an incubator at 50 °C for 4 h. The controls were grown under normal conditions. The cutoff criteria used to select the *ZmTHs* candidate genes was log2 |fold change| > 1.5.

### Chromosomal localization of trihelix family and intra-species collinearity analysis

The chromosome location and structural information of the trihelix family members were obtained from Maize GDB (https://qteller.maizegdb.org/). The intra-species covariance analysis was performed and visualized using TBtools software (Chen et al., 2020) to calculate the values of the duplicated genes with a non-synonymous/synonymous substitution ratio (Ka/Ks). The duplicated genes had greater than a 90% similarity.

### Protein characterization and subcellular localization analysis

Using the chromosomal localization information, we renamed the trihelix family members that responded to heat stress (*ZmTHs*). The protein properties were obtained using the ProtParam (https://web.expasy.org/protparam/) tool in Expasy. Plant-mPLoc (http://www.csbio.sjtu.edu.cn/bioinf/plant-multi/) and SOMPA (http://www.prabi.fr/) were used for the subcellular localization analysis prediction of protein secondary structure.

### Conserved motifs, gene structure and promoter analysis of trihelix family

Motif analyses were performed using the online tool MEME (http://memesuite.org/). TBtool software was used to analyze the exon-intron structures within the coding sequences (CDS) and the genomic sequences of each predicted *ZmTHs*. A total of 2,000 bp promoter sequences were obtained upstream of the CDSs using the sequence extraction tool in TBtools. These were submitted to the PlantCARE website (http://bioinformatics.psb.ugent.be/) for *cis*-acting elements analysis. The Gene Structure View tool in TBtools software was used for mapping.

### RNA isolation and qRT-PCR analyses of *ZmTHs* genes

Total RNA was extracted using RNAiso Plus (TaKaRa, Shiga, Japan), and cDNA was obtained by reverse transcription reaction using PrimeScript^TM^ RT reagent (with gDNA Eraser) (TaKaRa, Shiga, Japan). The solution was diluted 30 times and was stored at −20 °C for later use. Beacon Designer 8.1 was used to design *ZmTHs* specific primers according to their CDSs (Table 1) and the primers were synthesized using RuiBiotech.

**Table 1:** Primers used in the study.

ID	Forward primer (5′–3′)	Reverse primer (5′–3′)
*ZmActin1*	GGGATTGCCGATCGTATGAG	GAGCCACCGATCCAGACACT
*ZmTH01*	AACCGCAAACTGTAATGC	GACGACGATCTCCTTATTG
*ZmTH02*	CGCCACAAGATCGAGAAG	GGACTGGGACTTGGAAAA
*ZmTH03*	GTGGGAGAACATCAACAA	TGGTGGAAGTAAGGACAG
*ZmTH04*	CAGGAGGAGGAAGAAGTG	CCGAATTCCCGGATGATG
*ZmTH05*	GGAGAAGTGGGAGAACATC	TCCAGCTCGTCGAAGTAG
*ZmTH06*	GCGGCAATGCTAATAATGA	CTCGTCGTCCTGATCATC
*ZmTH07*	GAAGCATCTCAACTCCAA	CATCGTCATCATCAGAAAG
*ZmTH08*	TGCACGTTACAGAGAAGA	CTAGGATCTCAACCAGCTG
*ZmTH09*	GCAAGATTGATTCCTACC	CTCAGTATCGTTCTACCA
*ZmTH10*	ATCGGAGAGGGTTTCCTG	CTCCTCTGTGCATCAAGG
*ZmTH11*	ACAGCACATTGATGTCTG	CCGTGTTTCTTCTCTCATC

The qRT-PCR reaction system (15 μL) was comprised of 7.5 μL 2×TB Green Premix Ex Taq^TM^ II (TaKaRa, Shiga, Japan), 0.45 μL forward and reverse specific primers, 1.6 μL ddH_2_O, and 5 μL cDNA template. The reaction procedure was pre-denaturation at 95 °C for 30 s, denaturation at 95 °C for 5 s, 60 °C for 30 s, and 72 °C for 10 s for a total of 40 cycles. Each sample was repeated three times and the corresponding Ct values of different samples were detected using the CFX96 real-time PCR detection system (BioRad, Hercules, CA, USA). The relative expression of the target genes were calculated by the 2^−ΔΔCt^ method after standardization of the internal reference gene *ZmActin1* (Livak & Schmittgen, 2001). Data analysis was performed using GraphPad Prism and SPSS software.

## Results

### Identification of trihelix members and analysis of chromosome localization and intra-species collinearity

**Figure 1:**
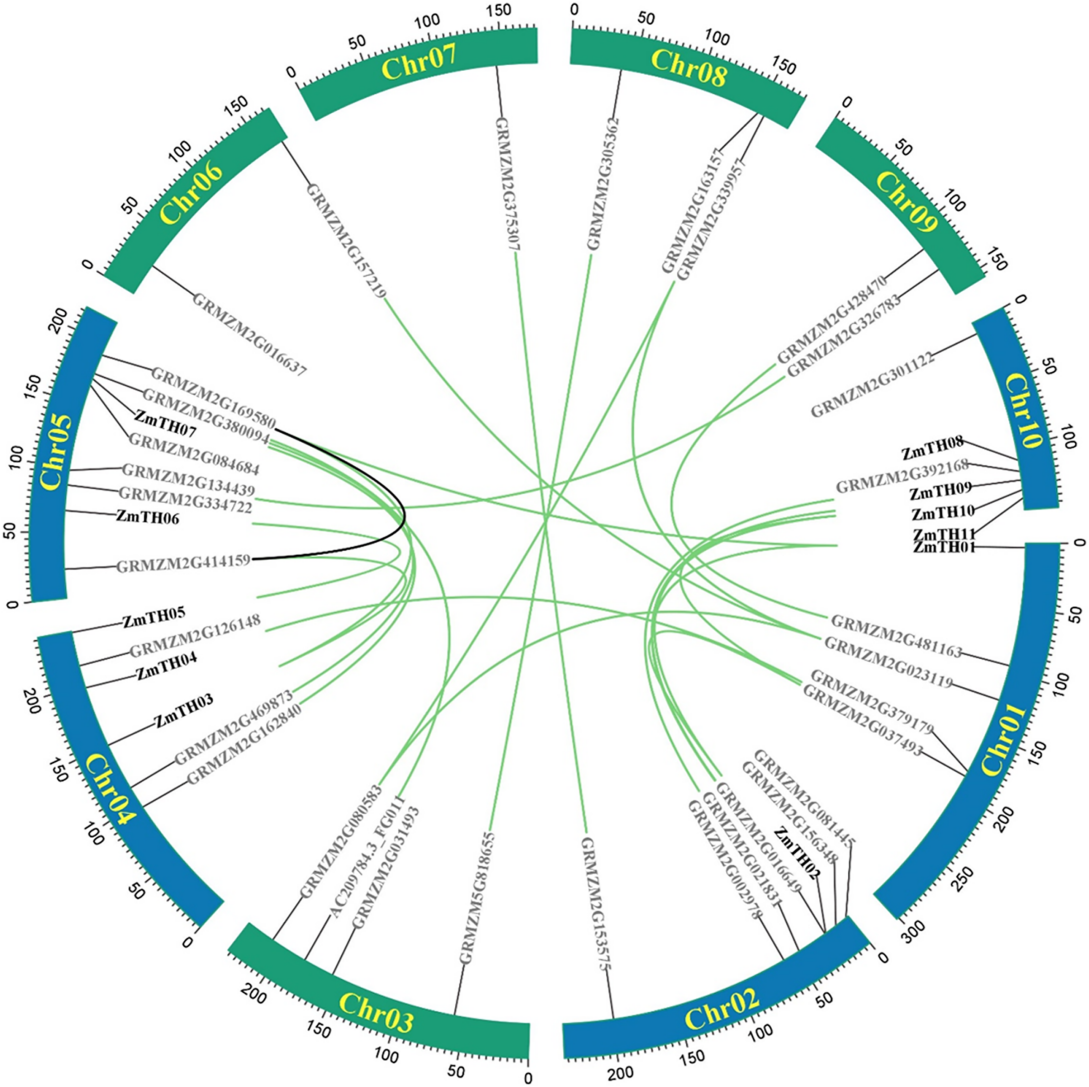
Distribution and intra-chromosomal segmental duplication map of trihelix genes in maize. The rectangles represent the ten chromosomes of maize on which the trihelix family genes are distributed. The blue rectangle indicates *ZmTHs* in response to heat stress. The black line inside the circles represent duplications of the trihelix gene family within the same chromosome. The green lines represent duplications between different chromosomes.

A total of 44 trihelix family members were identified in the maize genome, mainly on chromosomes Chr01, Chr02, Chr04, Chr05, and Chr10. Chr05 contained the greatest number, with eight trihelix family members. The remaining five chromosomes had a total of 12 trihelix genes; Chr07 had the smallest distribution with only one gene (Fig. 1). Intra-specific covariance analysis showed that there were 23 pairs of duplicated genes and replication events were the fundamental driving force of trihelix gene evolution. The Ka/Ks of duplicated trihelix gene pairs was less than one (Table 2), suggesting that the maize trihelix genes were subject to purifying selection pressure during evolution.

**Table 2:** Selection pressure analysis of the trihelix genes family.

No.	Gene 1	Gene 2	Ka	Ks	Ka/Ks
1	*ZmTH01*	GRMZM2G016649	0.37311	0.98202	0.37994
2	*ZmTH01*	GRMZM2G169580	0.42682	0.80630	0.52936
3	GRMZM2G481163	GRMZM2G428470	0.03630	0.26217	0.13848
4	GRMZM2G023119	GRMZM2G080583	0.26761	0.50898	0.52577
5	GRMZM2G023119	GRMZM2G157219	0.30478	0.90182	0.33796
6	GRMZM2G023119	GRMZM2G163157	0.31363	0.69083	0.45399
7	GRMZM2G379179	*ZmTH04*	0.04790	0.23494	0.20387
8	GRMZM2G037493	GRMZM2G156348	0.47282	0.55918	0.84555
9	GRMZM2G037493	*ZmTH11*	0.43272	0.78070	0.55427
10	GRMZM2G156348	*ZmTH11*	0.06091	0.21692	0.28078
11	*ZmTH02*	*ZmTH10*	0.02042	0.15092	0.13531
12	GRMZM2G021831	GRMZM2G392168	0.08172	0.19875	0.41116
13	GRMZM2G153575	GRMZM2G375307	0.04552	0.19888	0.22891
14	GRMZM5G818655	GRMZM2G305362	0.07718	0.18421	0.41894
15	AC209784.3_FGP011	GRMZM2G380094	0.24490	0.29264	0.83685
16	GRMZM2G080583	GRMZM2G163157	0.03650	0.19097	0.19112
17	GRMZM2G162840	GRMZM2G084684	0.05301	0.31890	0.16621
18	GRMZM2G469873	*ZmTH07*	0.04916	0.17069	0.28800
19	*ZmTH03*	GRMZM2G169580	0.09221	0.17291	0.53329
20	*ZmTH05*	*ZmTH06*	0.06205	0.13304	0.46641
21	GRMZM2G414159	*ZmTH03*	0.44651	0.83896	0.53222
22	GRMZM2G134439	GRMZM2G326783	0.08065	0.20586	0.39176
23	GRMZM2G169580	GRMZM2G414159	0.45678	0.83214	0.54893

### Analysis of heat stress expression profiles of trihelix members

**Figure 2:**
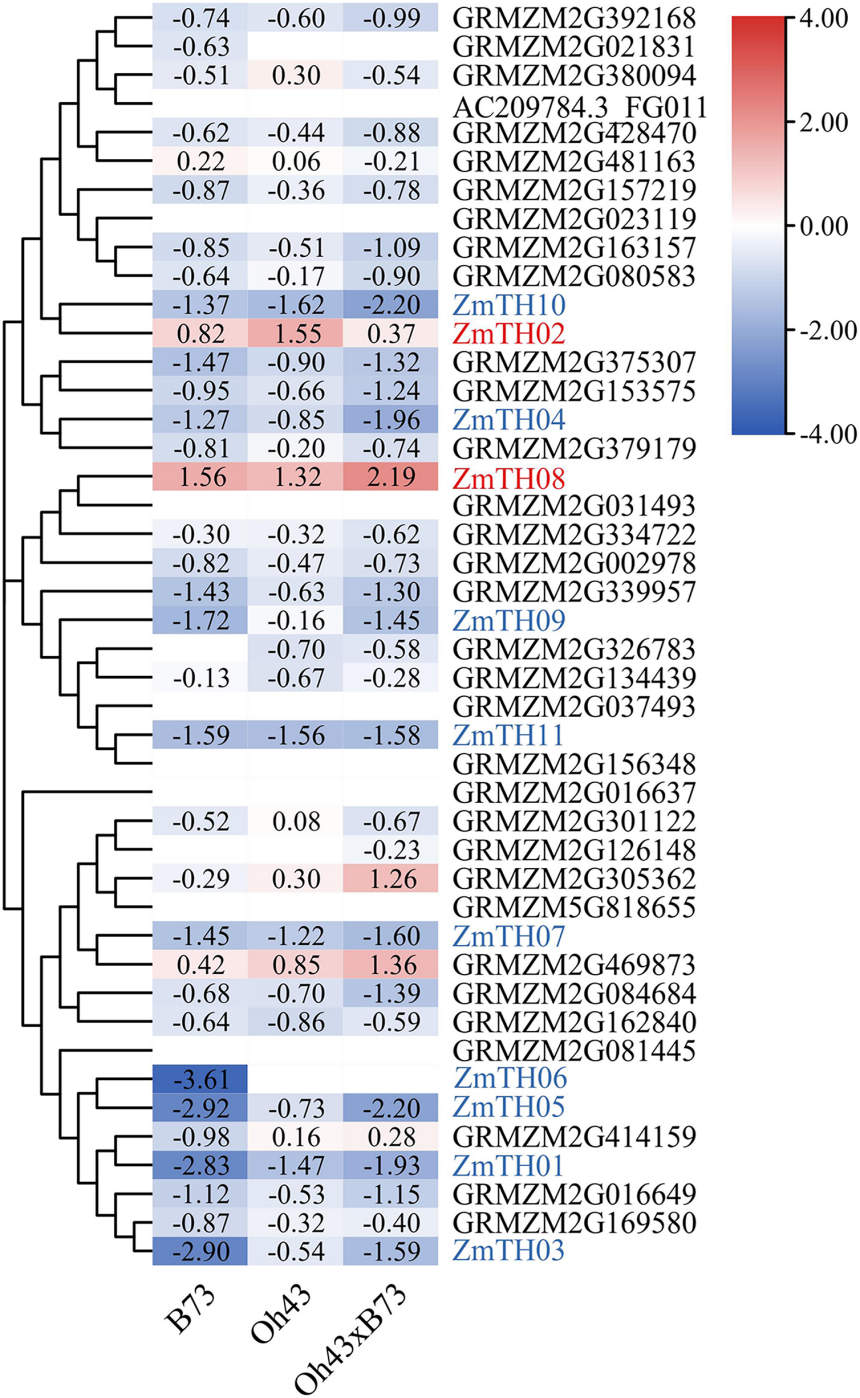
Heat map of trihelix genes expression profiles under heat stress. The red areas of the heat map indicate upregulated trihelix genes. The blue areas indicate downregulated trihelix genes with fold changes in the boxes.

The results of heat stress expression profiles of trihelix members are shown in Fig. 2. The up-regulation or down-regulation patterns of the trihelix genes in the aboveground tissues of the seedlings were similar between the parents (inbred lines B73, Oh43) and F1 (Oh43 × B73); most of them were down-regulated and only four genes were up-regulated. According to the expression log2 value, 11 trihelix transcription factors with a fold change greater than 1.5 were screened. These were named *ZmTH01*–*ZmTH11* based on the position of their respective genes on the chromosomes (Fig. 1). The expression profile showed that the expression of *ZmTH02* and *ZmTH08* were up-regulated while the remaining genes were down-regulated after heat stress treatment. These results suggest that *ZmTHs* may be involved in the regulation of heat stress response in plants.

### Evolutionary analysis of the trihelix family genes

**Figure 3:**
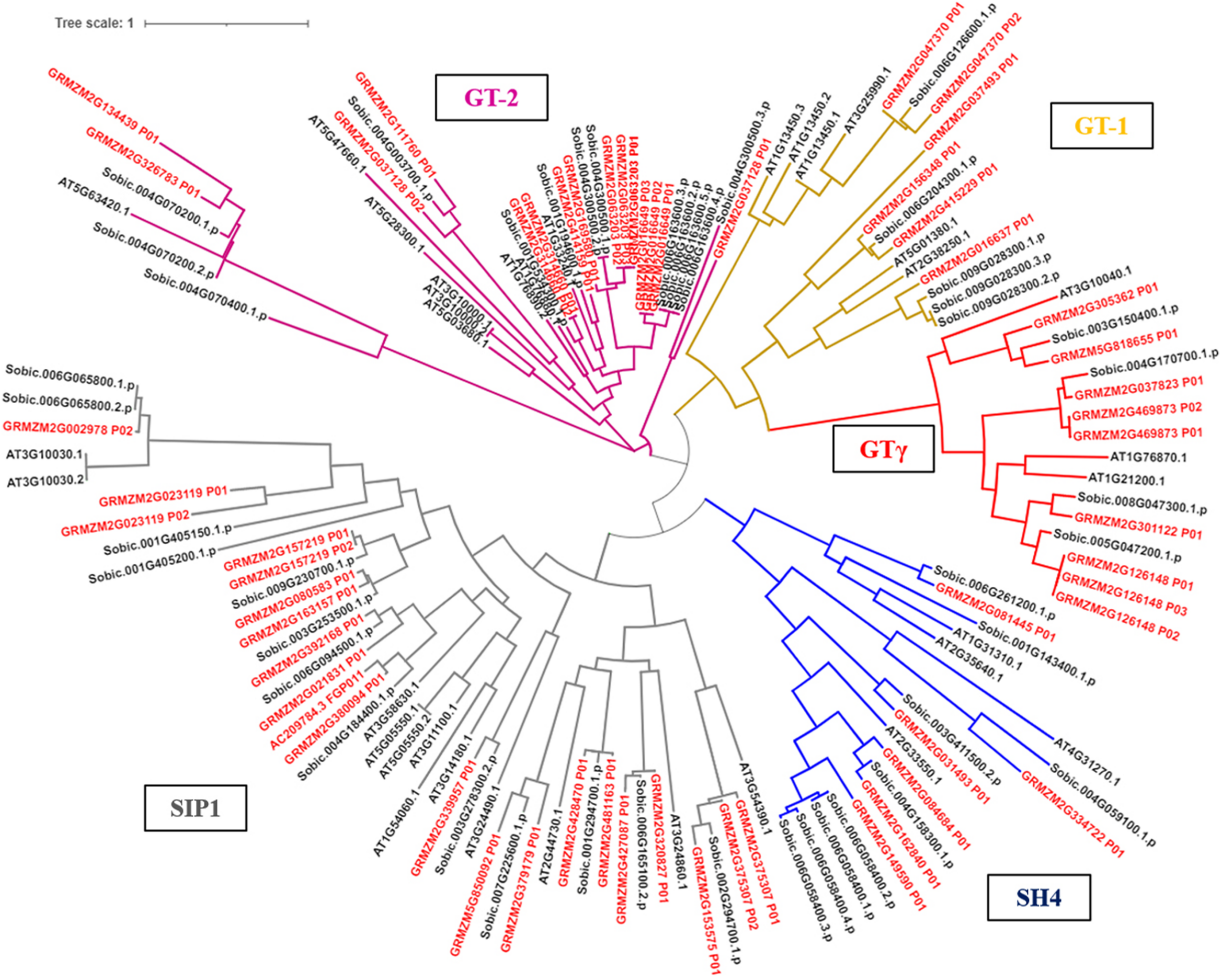
Phylogenetic analysis of the trihelix transcription factors among maize (Zm), sorghum (Sobic) and Arabidopsis (AT). The genes in red letters represent the trihelix family from maize and the genes in black letters represent the trihelix family from sorghum and Arabidopsis. Lines with different colors represent different subfamilies.

**Figure 4:**
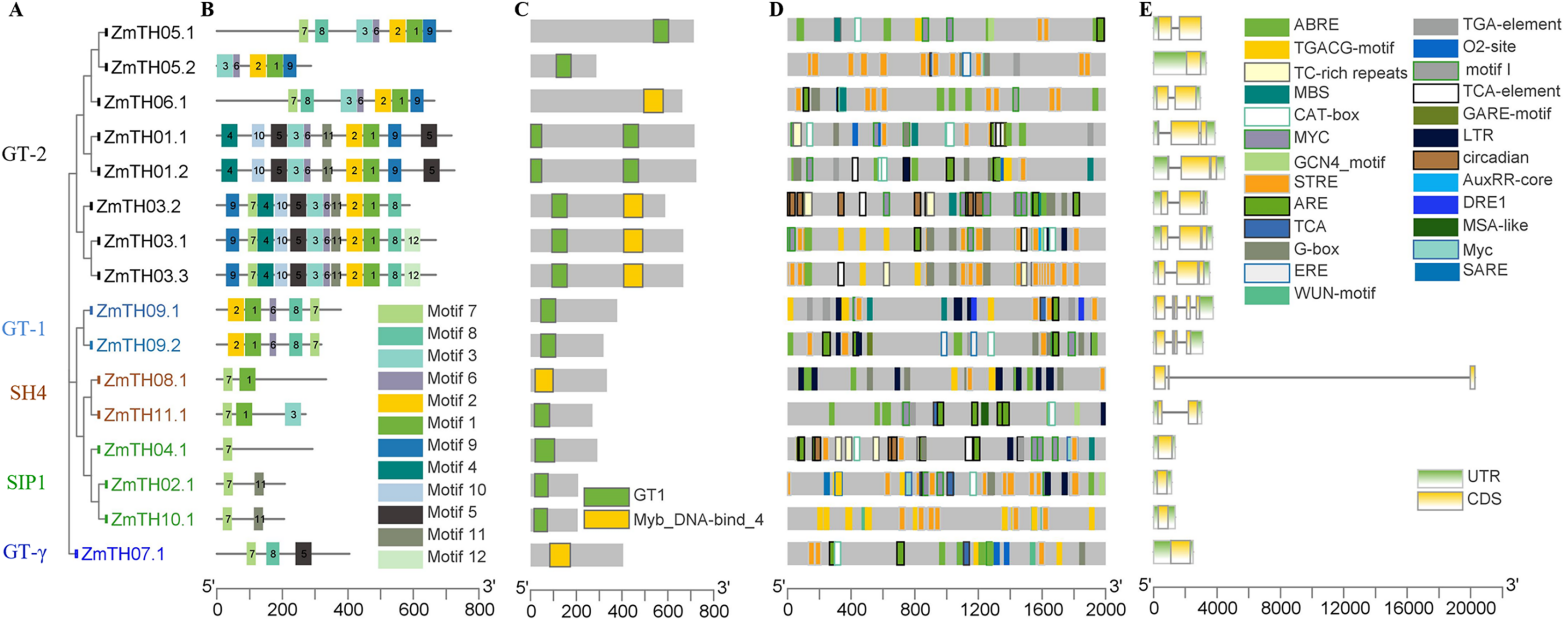
Analysis of the phylogenetic relationships, conserved motifs, promoter *cis*-acting elements, and exon-intron structures of trihelix family genes in maize. (A) The phylogenetic tree created by MEGA 7.0; the different colors of ZmTH represent different subfamilies. (B) Conserved motifs predicted in *ZmTH* proteins. MEME was used to identify the twelve motifs, with each number of the colored box representing a different motif. (C) Conservative domains found by CDD in *ZmTHs*. Rectangles with different colors represent different domains. (D) *Cis*-element analysis of *ZmTHs*. Boxes with different colors represent different *cis*-element identified by the PlantCARE, with each colored box representing a different motif, shown in the right. (E) Exon-intron structures were created using TBtool software. Yellow boxes, green boxes, and gray lines represent exons, UTR, and introns, respectively.

The phylogenetic tree results showed that the trihelix proteins of maize, Arabidopsis, and sorghum could be clearly classified into five subfamilies, and that the clustering branches of maize and sorghum were highly similar. The trihelix family members of maize were distributed in all five subfamilies, with the largest subfamily being SIP1. A total of 21 maize trihelix proteins were distributed; the GT-2 subfamily gathered 15, the GTγ subfamily distributed 9, and the GT-1 and SH4 subfamilies each contained six (Fig. 3). *ZmTHs* were also distributed in every subfamily though the number varied widely. GT-2 had the highest number with eight proteins, SIP1 contained three proteins, GT-1 and SH4 both contained two proteins, and GT-γ contained only one protein (Fig. 4A).

### Protein characterization and subcellular localization analysis of *ZmTHs*

*ZmTHs* genes were unevenly distributed on chromosomes 1, 2, 4, 5 and 10. Of these, chromosome 10 contained the highest number with four genes (Fig. 1). A total of 16 proteins were encoded, 15 of which were different (Table 3). The protein sequences and physicochemical properties of the *ZmTHs* transcription factors were quite different. The amino acid length varied from 206–725 amino acids, with the molecular weight ranging from 22.63–76.40 kD. The theoretical pI was 5.24–11.2 with eleven acidic proteins and five basic proteins. The instability index (II) of all *ZmTHs* proteins was greater than 40, indicating that they were unstable proteins. The grand average of hydropathicity was less than 0, suggesting that they belonged to hydrophilic proteins. Plant-mPLoc localization analysis showed that all *ZmTHs* proteins were localized in the nucleus. ZmTH04.1 was also distributed in chloroplasts (Table 3).

**Table 3:** The characteristics of *ZmTHs* proteins.

ID	cDNA name	Number of amino acids	Molecular weight	pI	Instability index	Grand average of hydropathicity	Subcellular localization
ZmTH01.1	GRMZM2G314660_T01	717	75,577.01	6.92	68.23	−0.745	Nucleus
ZmTH01.2	GRMZM2G314660_T02	725	76,401.90	6.73	68.05	−0.732	Nucleus
ZmTH02.1	GRMZM2G320827_T01	208	22,949.16	11.1	96.5	−0.682	Nucleus
ZmTH03.1	GRMZM2G063203_T01	668	71,709.60	5.78	55.45	−0.758	Nucleus
ZmTH03.2	GRMZM2G063203_T02	589	63,621.12	8.76	57.08	−0.804	Nucleus
ZmTH03.3	GRMZM2G063203_T03	668	71,709.60	5.78	55.45	−0.758	Nucleus
ZmTH04.1	GRMZM5G850092_T01	292	32,915.08	7.79	63.07	−0.794	Chloroplast, Nucleus
ZmTH05.1	GRMZM2G037128_T02	714	76,269.47	5.93	56.8	−0.862	Nucleus
ZmTH05.2	GRMZM2G037128_T01	288	31,441.32	5.24	45.03	−1.021	Nucleus
ZmTH06.1	GRMZM2G111760_T01	664	70,652.29	5.67	57.74	−0.796	Nucleus
ZmTH07.1	GRMZM2G037823_T01	405	46,187.92	5.91	47.34	−0.896	Nucleus
ZmTH08.1	GRMZM2G149590_T01	334	36,440.24	5.89	51.63	−0.794	Nucleus
ZmTH09.1	GRMZM2G047370_T01	379	41,935.71	6.28	48.46	−0.727	Nucleus
ZmTH09.2	GRMZM2G047370_T02	319	34,753.66	6.55	45.09	−0.692	Nucleus
ZmTH10.1	GRMZM2G427087_T01	206	22,628.80	11.2	96.72	−0.657	Nucleus
ZmTH11.1	GRMZM2G415229_T01	271	31,896.87	8.94	57.07	−1.204	Nucleus

### Secondary structure and conserved motifs analysis of *ZmTHs* proteins

The secondary structure of the proteins showed that the members of *ZmTHs* all contained four conformations. The highest proportion of conformations were the random coil and α-helix with an average of 90.51%. Among these two typical conformations, ZmTH02.1 had the highest percentage of 97.11%, and ZmTH09.1 had the lowest percentage, occupying 81.53%. In contrast, the proportion of extended chain and β-angle were relatively low, with an average of 10.49% (Table 4). Conservative motifs analysis showed that most of the closely related members in the phylogenetic tree had the same motifs. These results show that the protein architecture was remarkably conserved within a specific subfamily. MEME software showed that the motifs of *ZmTHs* of the GT-2 subfamily were the most complex, all members containing motif3, motif6, motif2, motif1, motif9, and the GT-1 subfamily contained motif2, motif1, motif6, motif8 and motif7. The SH4, SIP1 and GT-γ subfamily of the *ZmTHs* motifs were simpler and motif7 was widely distributed across all proteins (Fig. 4B). The conserved domain of *ZmTHs* was GT1 or Myb_DNA-bind_4 (Fig. 4C), which was a typical triple helix structure. Five proteins in the GT-2 subfamily contained two triple helix structures, while the remaining had only one (Fig. 4C).

**Table 4:** Secondary structure of *ZmTHs* proteins.

ID	α-helix	Extended strand	β-turn	Random coil
ZmTH01.1	34.73%	8.65%	5.44%	51.19%
ZmTH01.2	34.34%	8.41%	5.93%	51.31%
ZmTH02.1	52.40%	1.92%	0.96%	44.71%
ZmTH03.1	40.12%	6.29%	4.34%	49.25%
ZmTH03.2	39.73%	5.26%	3.90%	51.10%
ZmTH03.3	40.12%	6.29%	4.34%	49.25%
ZmTH04.1	47.60%	1.37%	1.71%	49.32%
ZmTH05.1	34.59%	6.86%	5.88%	52.66%
ZmTH05.2	57.99%	2.08%	3.82%	36.11%
ZmTH06.1	35.69%	6.02%	5.27%	53.01%
ZmTH07.1	51.60%	3.46%	4.20%	40.74%
ZmTH08.1	45.51%	4.19%	5.39%	44.91%
ZmTH09.1	35.36%	12.40%	6.07%	46.17%
ZmTH09.2	34.80%	10.34%	4.70%	50.16%
ZmTH10.1	51.94%	3.40%	0.00%	44.66%
ZmTH11.1	63.84%	0.74%	2.21%	33.21%

### Gene structure and stress-related *cis*-elements analysis of *ZmTHs*

The gene structures of *ZmTHs* were quite different and alternative splicing were observed in Fig. 4E. Among them, *ZmTH5.2* of the SIP1, GTγ, and GT-2 subfamilies, contained only one segment of exons with 5′ UTR and 3′ UTR and no introns. The remaining of *ZmTHs* members all contained introns, and two or more exons, with 5′ UTR or 3′ UTR. The most remarkable feature of *ZmTH08.1* was that it contained an extra-long segment of introns of approximately 20,000 bp, which may be related to the regulation of genes expression (Han et al., 2016).

We analyzed the stress-related *cis*-elements in their promoter regions using the PlantCARE database to better understand the potential regulatory mechanisms of *ZmTHs* in heat stress. The results showed that there were various stress response *cis*-elements in *ZmTHs* promoters, such as high temperature (STRE, TCA), low temperature (LTR), drought (MBS, DRE1), anaerobic (ARE) and wounding (WUN-motif). STRE high temperature stress response elements were predominant, with an average of six per promoter region. In addition, hormone response elements such as salicylic acid, gibberellin, abscisic acid, jasmonic acid, methyl jasmonate, auxin and ethylene were also found in the promoter regions of *ZmTHs*. There were also tissue-specific expression elements such as meristem, endosperm and root, as well as elements involved in the regulation of maize alcohol protein metabolism (Fig. 4D). These results suggest that the expression of *ZmTHs* may be regulated by various environmental factors and have different expression specificities in different tissues.

### Tissue‑specific expression analysis of *ZmTHs* at the flowering stage

**Figure 5:**
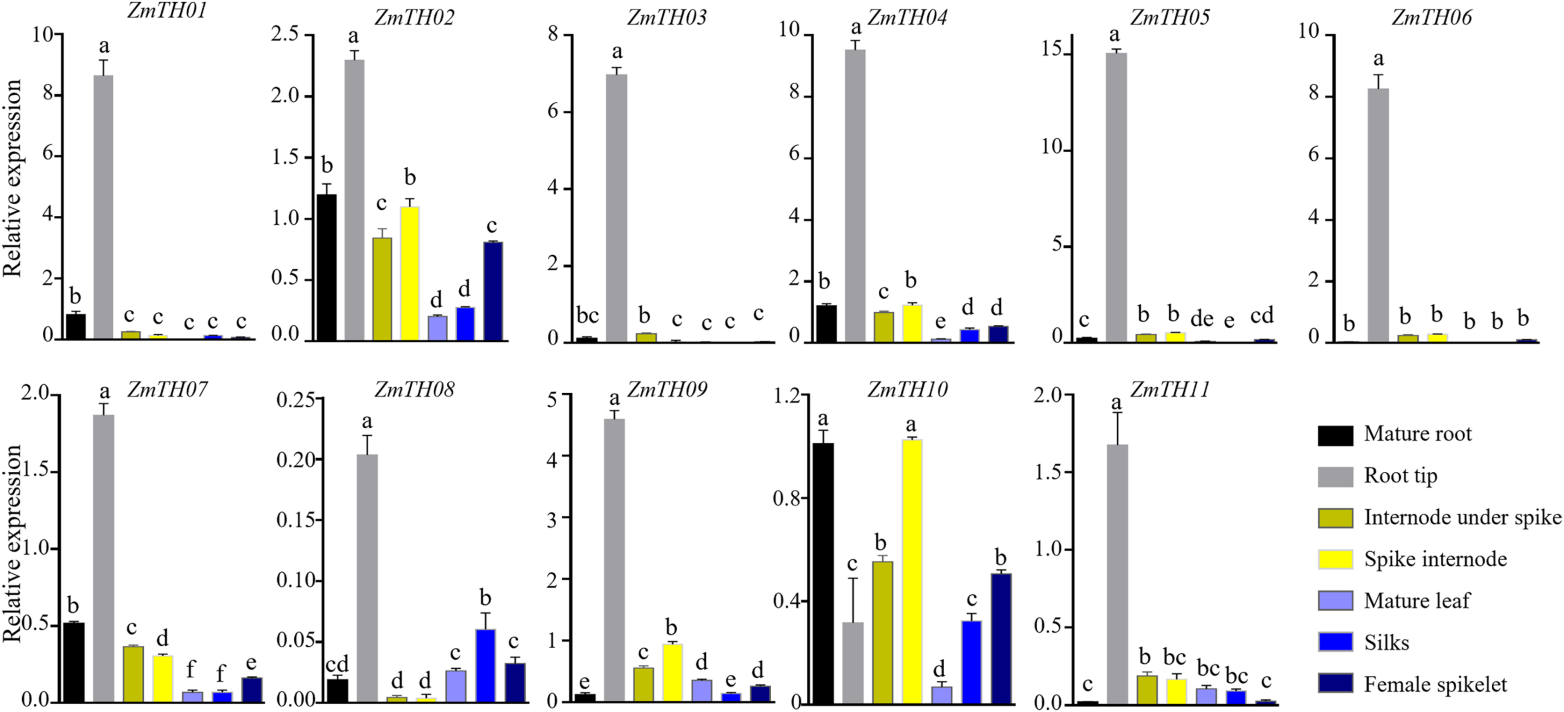
The expression profiles of the *ZmTHs* genes in different tissues. B73 seedlings grown during flowering stage were harvested for RNA extraction and qRT-PCR was used to detect the expression of *ZmTHs*. *ZmActin1* was used as an internal standard. The bars indicate the mean ± SD of three independent measurements. Lowercase letter(s) above the bars indicate significant differences (α = 0.05, Duncan’s multiple range tests) among the tissues.

The expression levels of *ZmTHs* in the mature root, root tip, spike internode, internode under spike, mature leaf, silks and female spikelet of B73 grown under normal conditions during the flowering stage are shown in Fig. 5. The GT-2 subfamily members, including *ZmTH01*, *ZmTH03*, *ZmTH05* and *ZmTH06*, were mainly expressed in roots, especially in the root tip, where the expression was seven to 15 times higher than in *ZmActin1*. *ZmTH02*, *ZmTH04*, *ZmTH07*, *ZmTH09*, *ZmTH10* and *ZmTH11*. These genes mostly belong to the GT-1, GTγ and SIP1 subfamilies and had relatively high expressions in the internode. The expression levels of SIP1 subfamily members, including *ZmTH02*, *ZmTH04* and *ZmTH10*, were higher in female spikelets. The relative expression of the SH4 subfamily members, namely *ZmTH08* and *ZmTH11*, were lower in all tissues except the root tip and were less than 20% of *ZmActin1* (Fig. 5). These results were consistent with the fact that the promoter regions of *ZmTHs* contain root- and meristem-specific expression *cis*-elements. These results revealed that the expression levels of each subfamily member in different tissues were quite different.

### Analysis of expression profiles of *ZmTHs* under heat and drought stress by qRT-PCR

**Figure 6:**
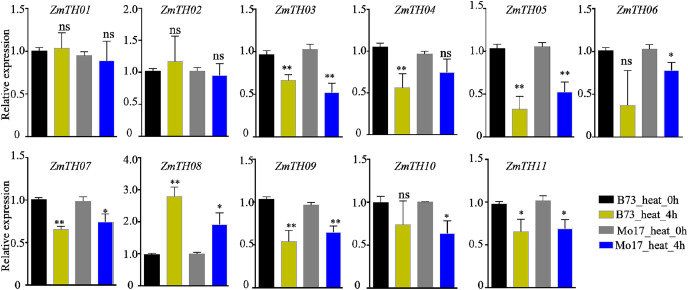
The relative expression of *ZmTHs* under heat stress in maize. Untreated plants were used as a control and *ZmActin1* was used as an internal standard. The bars indicate the mean ± SD of three independent measurements. One asterisk (*) and two asterisks (**) represent *p* < 0.05 and *p* < 0.01, respectively. ns indicates no significant differences (Student’s *t*-tests).

The expression levels of *ZmTHs* under heat stress as detected by qRT-PCR are shown in Fig. 6. The results showed that *ZmTH08* was up-regulated by heat stress, while *ZmTH03*, *ZmTH04*, *ZmTH05*, *ZmTH06*, *ZmTH07*, *ZmTH09*, *ZmTH10* and *ZmTH11* were all down-regulated by heat stress in inbred lines B73 and Mo17. These results were consistent with the expression profiles of the *ZmTHs in silico*. However, *ZmTH01* and *ZmTH02* were slightly up-regulated in B73 by treatment at 42 °C for 4 h. These results were not identical to the bioinformatics-predicted results of the regulation pattern in leaves treated at 50 °C for 4 h, which were slightly down-regulated by heat stress in Mo17 inbred line. The gene expression patterns of *ZmTHs* were not completely consistent in various materials treated at different temperatures. These results suggested that *ZmTHs* respond to heat stress and may have potential functions in abiotic stress.

**Figure 7:**
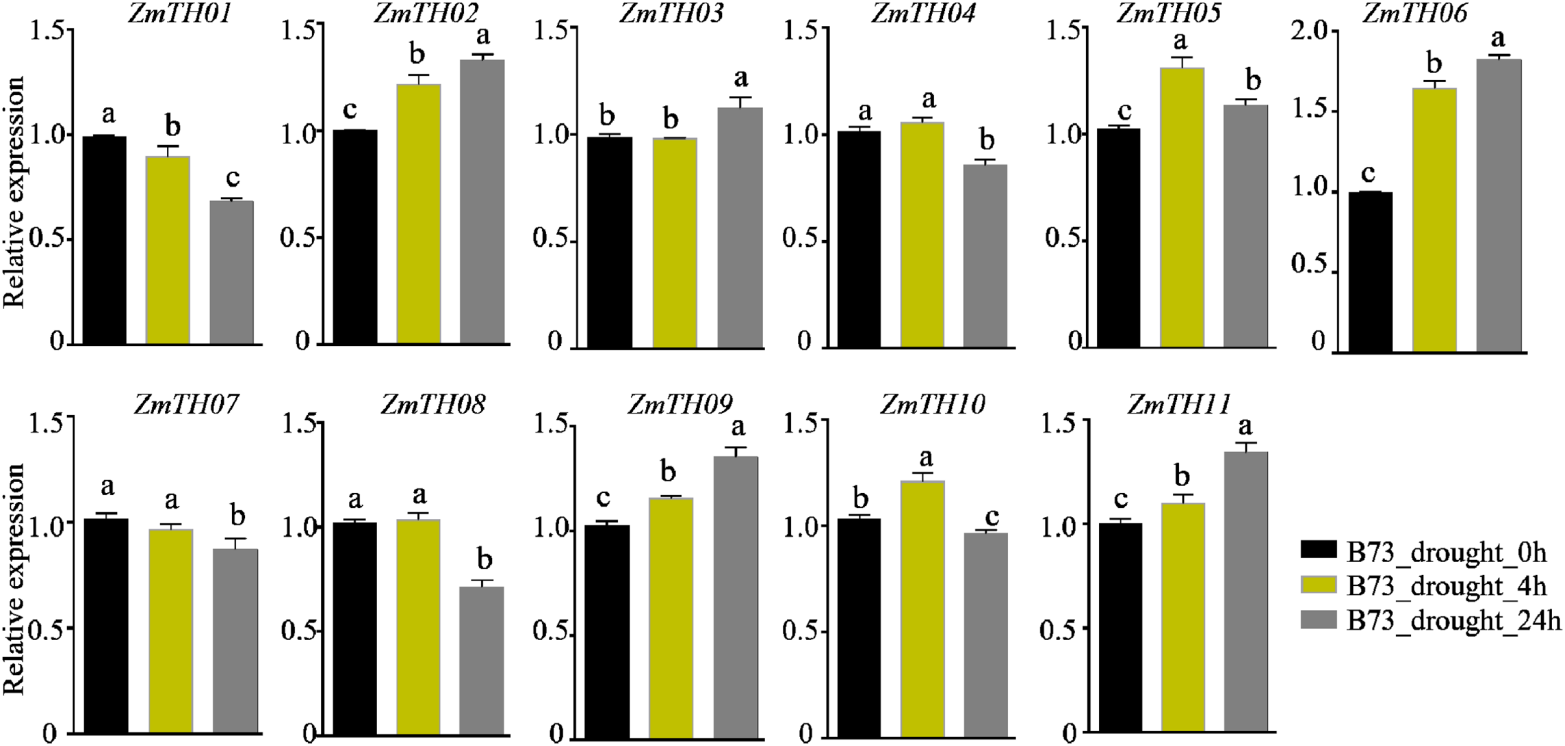
The relative expression of *ZmTHs* under drought stress in maize. Untreated plants were used as a control and *ZmActin1* was used as an internal standard. The bars indicate the mean ± SD of three independent measurements. Lowercase letters denote significant differences as determined by Duncan’s at 0.05 level.

The analysis of promoter *cis*-acting elements revealed that *ZmTHs* contained abundant drought stress response elements, such as MBS and DRE1. qRT-PCR was used to detect the expression of *ZmTHs* genes under drought stress and the results showed that *ZmTH02*, *ZmTH05*, *ZmTH06*, *ZmTH09*, and *ZmTH11* were up-regulated by drought stress in the inbred line B73. *ZmTH06* was especially affected and had expression levels nearly twice those of the untreated genes. The expressions of *ZmTH01* and *ZmTH07* were down-regulated by drought stress. The expression patterns of the remaining *ZmTHs* genes subjected to 4 h and 24 h drought treatments were inconsistent. For instance, the expression of *ZmTH04* and *ZmTH08* were almost unchanged after 4 h drought treatment compared with the control. However, after 24 h of drought treatment, the expression of both trended downward (Fig. 7). These results indicated that *ZmTHs* may have potential functions in drought stress.

## Discussion

Transcription factors are DNA-binding proteins that interact with *cis*-elements in the promoter regions of target genes and regulate their expression. The trihelix family of transcription factors has important roles in plant growth and development, and biotic and abiotic stress responses (Kaplan-Levy et al., 2012). Currently, trihelix factors have been identified in many dicot and monocot plants, such as Arabidopsis (Kaplan-Levy et al., 2012), rice (Li et al., 2019), soybean (Liu et al., 2020a), wheat (Wang et al., 2019), sorghum (Li et al., 2021a) and cotton (Mo et al., 2019). In this study, 44 members of the trihelix transcription factors were obtained from the whole maize genome (Fig. 1), which is consistent with the number of trihelix genes identified in other maize research (Jiang et al., 2020). A phylogenetic tree was constructed based on the trihelix protein sequences, and the maize trihelix family members were classified into five subfamilies (Fig. 3). These classifications were similar to the evolutionary relationship classification of trihelix in Arabidopsis (Kaplan-Levy et al., 2012). The Ka/Ks ratio may determine the evolutionary pressure of genes (Hurst, 2002). The Ka/Ks values of the trihelix genes in maize were all less than one, indicating that the purifying selection played an important role in evolution (Table 2). This result is consistent with the selective pressure on trihelix genes during the evolution of soybean (Liu et al., 2020a). Many trihelix genes were responsive to heat stress in brassica and sorghum (Li et al., 2021a; Zhang et al., 2022). Genevestigator and the NCBI platform are powerful bioinformatics tools for genes functional analysis (Camacho et al., 2009; Hruz et al., 2008). We identified 11 genes based on heat stress expression profiles in maize and named them *ZmTH01-11* (Figs. 1 and 2). These genes may play vital roles in heat stress.

The protein characteristics varied greatly among the members of *ZmTHs* (Table 3), which was consistent with the protein conservation within the subfamily branches and greater variability between branches. All of the trihelix genes were predicted to be in the nucleus, except *ZmTH04* which localized to the chloroplast, which indicated that *ZmTH04* may have functions in chloroplasts. The trihelix transcription factors in soybean, rice and sorghum were also localized in the chloroplast (Li et al., 2019; Li et al., 2021a; Liu et al., 2020a). *GRY79*, encoding the metal-beta-lactamase triple helix chimera, is reportedly involved in chloroplast development at seedling stage in rice (Wan et al., 2015). Gene structure analysis showed that the number of exons in CDS was 1–4 and the number of introns was 0–3 (Fig. 4), indicating that the gene structure of this family was relatively simple. This was similar to the findings of research on the trihelix transcription factor family in Arabidopsis, sorghum and rice (Kaplan-Levy et al., 2012; Li et al., 2019; Li et al., 2021a). Interestingly, intron-free genes were found in the SIP1 and GTγ subfamilies (Fig. 4), which was previously observed in *Brassica napus and* sorghum (Li et al., 2021a; Zhang et al., 2022). The high proportion of α-helices in the secondary structure of *ZmTHs* (Table 4) was consistent with α-helices being a constituent component of the typical trihelix structure (Lam, 1995). Random coiling is an important region for the functional implementation and conformation of the protein molecule (Mittal et al., 2018). Therefore, the functions of *ZmTHs* members are complex. Conserved motif analysis showed that the members within each subfamily of *ZmTHs* were similar and relatively conserved in structure, but the motif structures differed greatly between different subfamilies (Fig. 4). These results were similar to those found in soybean (Liu et al., 2020a), suggesting that the five subfamilies may have different functions in maize.

Promoter *cis*-elements play important roles in plant development and their response to biotic and abiotic stresses (Priest, Filichkin & Mockler, 2009). The promoter regions of *ZmTHs* were rich in tissue-specific expression and abiotic stress response elements (Fig. 4). Therefore, we detected the expression patterns of these genes in tissues and abiotic stress conditions using qRT-PCR. Trihelix genes have had reported involvement in the development of plant organs (Kaplan-Levy et al., 2012; Luo, Zhao & Lu, 2012). *ZmTHs* gene expression had noticeable tissue specificity, which was similar to rice (Li et al., 2019). GT-2 subfamily members were mainly expressed in roots, especially in the growing zone of roots. This is consistent with the expression profiles of *TaGTs* in wheat (Wang et al., 2019). The GT-1, GTγ and SIP1 subfamilies had a relatively high expression in the maize internode. The SIP1 subfamily had a high relative expression in the female spikelet, while the SH4 subfamily members had low relative expressions in all detected tissues (Fig. 5). These genes may play roles during the growth and development in the corresponding tissues, however, there need to be further experiments to verify their functions. The expression patterns of the trihelix genes under abiotic stresses such as heat and drought have been reported (Li et al., 2021a; Zhang et al., 2022). *GT-1* activates the expression of *HsfA2* through directly binding it promoter, which in turn drives the expression of downstream target genes to enhance plant heat tolerance under heat stress (He et al., 2022). The expression of *ZmTH08* was up-regulated in B73 and Mo17 inbred lines after heat stress treatment at 42 °C, while the most of *ZmTHs* were down-regulated in B73 and Mo17 by heat stress (Fig. 6). This suggested that most *ZmTHs* may play a negative role under heat stress. The genes’ expression pattern, however, may vary in different tissues. For example, most of the *SbTHs* were up-regulated in sorghum stems, while they were down-regulated in the leaves after a 2 h heat treatment (Li et al., 2021a). Therefore, a more refined experimental validation is needed. The expression of *ZmTH06* was significantly up-regulated by drought stress in B73 seedlings, while *ZmTH01* and *ZmTH07* were down-regulated (Fig. 7). These results were consistent with the abundant abiotic stress-responsive *cis*-acting elements contained in the promoter regions of *ZmTHs* (Priest, Filichkin & Mockler, 2009). The subfamily members of *ZmTHs* may be involved in regulating the development of different tissues and organs in response to abiotic stresses, which affect the growth of maize. However, their specific roles in the stress response need to be further investigated.

## Conclusion

A total of 44 trihelix transcription factors were obtained from the whole maize genome. A total of 11 genes of the trihelix family were named *ZmTH01*-*11* in response to their reaction under heat stress. Phylogenetic analysis classified the *ZmTHs* into five subfamilies. These subfamilies differed greatly in protein and gene structure but were otherwise highly similar within the subfamilies. The promoter regions in *ZmTHs* contained tissue-specific expression *cis*-elements, which were differentially expressed in different tissues of maize. The expression of *ZmTH08* was up-regulated by heat stress and *ZmTH03*, *ZmTH04*, *ZmTH05*, *ZmTH06*, *ZmTH07*, *ZmTH09*, *ZmTH10* and *ZmTH11* were down-regulated by heat stress in both the B73 and Mo17 inbred lines. *ZmTH06* was up-regulated by drought stress, while *ZmTH01* and *ZmTH07* were down-regulated in the B73 line. This research will lay a foundation for further analyzing the biological functions of the maize trihelix members in responding to abiotic stresses.

## Supplemental Information

10.7717/peerj.15312/supp-1 Supplemental Information 1 Supplemental figures and raw data. Plant phenotype before and after heat stress or drought treatment

## Additional Information and Declarations

### Data Availability

The following information was supplied regarding data availability: The raw measurements are available in the Supplemental File.
